# Long-term voice evaluation after arytenoid adduction surgery in patients with unilateral vocal fold paralysis

**DOI:** 10.1007/s00405-023-08165-9

**Published:** 2023-08-16

**Authors:** Kenichi Watanabe, Ai Hirano, Yuta Kobayashi, Takeshi Sato, Yohei Honkura, Yukio Katori

**Affiliations:** 1 Department of Otolaryngology, Tohoku Rosai Hospital, 4-3-21 Dainohara, Aoba-ku, Sendai, Miyagi 981-8563 Japan; 2 Department of Otolaryngology, Head and Neck Surgery, Tohoku University Graduate School of Medicine, 1-1 Seiryo-Machi, Aoba-ku, Sendai, Miyagi 980-8574 Japan

**Keywords:** Unilateral vocal fold paralysis, UVFP, Arytenoid adduction, Laryngeal framework surgery, Voice Handicap Index, VHI

## Abstract

**Purpose:**

Laryngeal framework surgery, including medialization laryngoplasty and arytenoid adduction (AA), is expected to have a lasting or permanent effect in patients with unilateral vocal fold paralysis (UVFP); however, there are few reports about the long-term outcomes of AA. This study aimed to evaluate the long-term postoperative effects of AA surgery and examine its stability and reliability.

**Methods:**

This study collected the voice handicap index (VHI) questionnaire from patients with UVFP who underwent AA more than 2 years previously. The VHI values preoperatively and 3 months postoperatively (early postoperative evaluation) were retrospectively calculated, and VHI values more than 2 years after surgery (late postoperative evaluation) were collected by mailing a sheet to the patients and asking to fill and return it. Possible influenced subscales such as age, sex, causes of UVFP, affected side, and surgeons were also analyzed.

**Results:**

A total of 77 patients with UVFP who underwent AA had significantly lower early and late postoperative evaluations than preoperative evaluations. In 38 patients with no missing values, there were no significant differences between early and late postoperative evaluations, measured at a median of approximately 5 years. There were also no significant differences between early and late postoperative evaluations in any of the subscale groups.

**Conclusion:**

Patients with UVFP who underwent AA surgery achieved stable voice improvement in the long term after surgery.

## Introduction

Unilateral vocal fold paralysis (UVFP) is a common complication encountered by otolaryngologists. Incomplete glottal closure caused by UVFP can lead to mild to severe breathy hoarseness, poor cough reflex, dysphagia, and aspiration. Some authors have reported that patients with UVFP showed a higher voice handicap than those with other types of organic dysphonia, such as laryngeal nodules, polyps, or cysts [1, 2]. Several interventional options have been developed for the treatment of UVFP, including medialization laryngoplasty (ML), injection laryngoplasty (IL), arytenoid adduction (AA), and laryngeal reinnervation. Among these interventions, AA is notable in that it can medialize and close the large posterior glottic gap. Moreover, it can correct vocal fold height mismatch by adducting and rotating the arytenoid cartilage. In addition, to compensate for atrophic change or bowing of the membranous portion, AA in combination with ML has recently been recommended by several clinicians [3–6]. For example, Slavit et al. concluded that the AA procedure could correct a large posterior glottic gap and that AA combined with ML is an effective technique in patients with marked vocal cord bowing [4]. McCulloch et al. evaluated the voice outcomes of patients who underwent ML and ML-AA and showed significant improvement in multiple voice parameters [5]. Mortensen et al. reported that AA could correct the physiology of an incompetent larynx better than ML alone, resulting in a statistically greater degree of change in acoustic and aerodynamic parameters [6]. Recently, Sano et al. showed that ML using titanium implants combined with AA resulted in significant improvements in phonation function, subjective voice quality, and patient self-assessment [7].

While voice improvement by IL is considered short- or medium-term, depending on the nature of the injected substance, laryngeal framework surgery, including ML and AA, is expected to have a lasting or permanent effect after surgery [8, 9]. However, evidence supporting the long-term consistency of laryngeal framework surgery has not yet been clearly elucidated. Several authors have reported the long-term stability of ML using silastic block [10], titanium [11], and Montgomery^®^ [12] implants. However, other authors mentioned the fading effect of ML over a period of years [13, 14]. As for AA, there are few reports on long-term postoperative voice function, with the outcome of voice measurements, including aerodynamic and acoustic measures and subjective voice quality assessment, showing comparable results between 3 and 24 months after AA ± ML by Hassan et al. [15]. Currently, there is a lack of long-term evaluation of the AA more than 2 years after surgery.

In this study, we aimed to evaluate the long-term postoperative effects of AA in patients with UVFP and investigate its stability and reliability. We collected the voice handicap index (VHI) questionnaire from patients with UVFP who underwent AA at least 2 years previously. These long-term postoperative data were compared with the previously acquired preoperative and short-term postoperative VHI values.

## Materials and methods

### Study design and participants

Patients with UVFP who underwent AA more than 2 years previously at the Department of Otolaryngology, Head and Neck Surgery, Tohoku University Hospital, were included in this study. Exclusion criteria included age < 20 years and incomplete surgery (e.g., laryngeal scarring). Seventy-seven patients were enrolled between 2014 and 2020, excluding one who spontaneously recovered from vocal fold paralysis after surgery.

The VHI values preoperatively and approximately 3 months postoperatively (early postoperative evaluation) were retrospectively calculated from the medical records. In addition, VHI values more than 2 years after surgery (late postoperative evaluation) were collected by mailing a sheet to the patients and asking them to fill and return it. Patients confirmed that they filled out the questionnaire. Furthermore, patients were asked to disclose possible further voice surgeries.

Once the data were obtained, the VHI values of the late postoperative evaluation were compared with those of the early postoperative evaluation. Furthermore, five subscales, including age differences, sex, causes of UVFP, affected side, and surgeons, were analyzed. The groups divided into two or three within each subscale were compared. The groups were compared during the early and late postoperative evaluations.

### Examination of perceptual voice evaluation

#### Voice Handicap Index

The VHI is a 30-item self-administered questionnaire developed by Jacobson et al. to quantify a patient’s perception of disability resulting from a voice disorder [16]. The Japanese version of the VHI was used in our study, with scores ranging from 0 to 120.

### Surgery

The technique was performed using previously published methods [5, 17] with some modifications as follows. First, the affected side of the thyroid cartilage lamina was exposed. The cricothyroid joint was not routinely dislocated. After determining the muscular process of the arytenoid cartilage, nylon threads were sutured to the muscular process, pulled toward the lateral cricoarytenoid muscle, and fixed to the cricothyroid ligament to perform the AA. Next, a window for the ML was created at the vocal-fold level in the thyroid cartilage lamina. The decision to preserve or remove the cartilage window depended on the surgeon’s choice. Gore-Tex^®^ was fashioned into a single 7- to 8-mm-wide ribbon, layered, and placed into the sub-perichondrial space beneath the window as the implant.

### Statistical analysis

Friedman’s test was used to analyze the evolution of the VHI over time (preoperatively, early postoperatively, and late postoperatively). The Wilcoxon signed-rank test was used to compare the late and early postoperative evaluations. We conducted the Mann–Whitney *U* or Kruskal–Wallis tests to compare the groups within the subscales and the Wilcoxon signed-rank test to compare the groups in the early and late postoperative evaluations. Statistical analyses were performed using EZR version 1.61 (Saitama Medical Center, Jichi Medical University, Saitama, Japan) [18].

## Result

From the medical records, 11 patients were found dead or in terminal condition. None of the deaths were related to AA. The 66 remaining patients were asked to participate in this study by mailing the VHI questionnaire along with an accompanying letter and an informed consent form. The overall response rate was 63.6% (42/66). The death of one patient was reported by a relative.

**Fig. 1:**
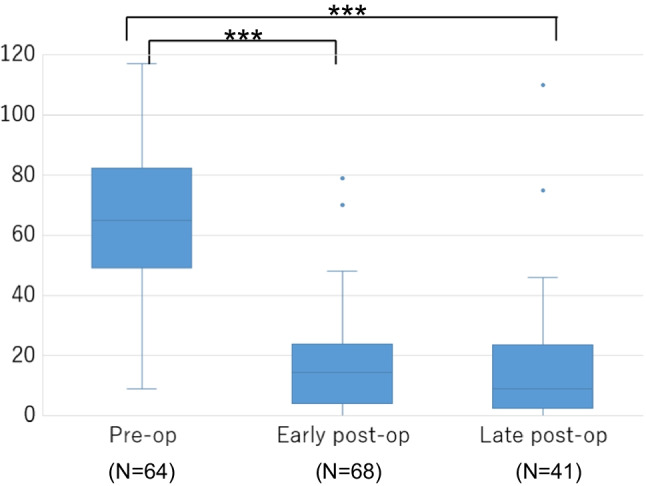
Evolution of VHI over time in all patients. VHI, voice handicap index

We collected the VHI values for 64 patients preoperatively, 68 early postoperatively, and 41 late postoperatively from a total of 77 patients. Figure 1 displays boxplots showing the evolution of VHI over time in all patients. The patients showed significantly lower VHI in the early postoperative evaluation than that in the preoperative evaluation (*P* = 0.0000017) and significantly lower in the late postoperative evaluation (*P* = 0.0000017). The median preoperative VHI was 65, whereas 14.5 in the early postoperative evaluation and 9 in late postoperative evaluations.

For the comparison of early and late postoperative evaluations, only 38 patients with no missing values in the two groups were selected. Table 1 shows the demographic and clinical characteristics of patients. The median age of the patients was 66.0 years; 31 were men and 7 were women. The affected side was the left for 29 patients and the right for 9. The most frequent cause of UVFP was thoracic aortic aneurysm (TAA) (36.8%), followed by lung cancer (18.4%) and thyroid surgery (15.8%). Most AA surgeries are performed in combination with ML, which is also known as thyroplasty type 1 (TP1). Four surgeons performed these surgeries. The median follow-up duration of the late postoperative evaluation was 61 months (31–101 months). Four patients underwent further voice surgeries after AA: two received collagen injections, one received an autologous fat injection, and one underwent re-ML.

**Table 1:** The demographic and clinical characteristics of patients (*N* = 38)

Demographic	*n* (%)
*Sex*	
Male	31 (81.6)
Female	7 (18.4)
*Age*	
Median 66.0 y.o. (32–80 y.o.)	
*Side of UVFP*	
Left	29 (76.3)
Right	9 (23.7)
*Etiology of UVFP*	
Surgery of thoracic aortic aneurysm	14 (36.8)
Lung cancer	7 (18.4)
Thyroid surgery	6 (15.8)
Idiopathic	3 (7.9)
Jugular foramen tumor	2 (5.3)
Esophageal cancer	2 (5.3)
Others	4 (10.5)
*Combined with*	
TP1	37 (97.4)
AA alone	1 (2.6)
*Surgeons*	
A	23 (60.5)
B	7 (18.4)
C	6 (15.8)
D	2 (5.3)

UVFP, unilateral vocal fold paralysis; TP1, thyroplasty type 1; AA; arytenoid adduction surgery

**Fig. 2:**
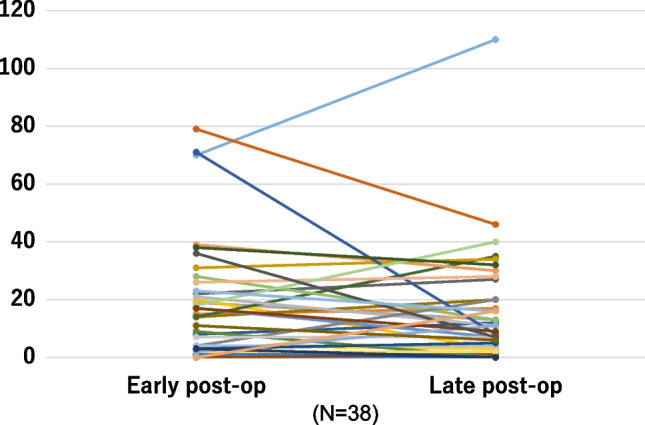
Comparison between early and late postoperative VHI. VHI, voice handicap index

Figure 2 displays a linear graph comparing the VHI values in the early and late postoperative evaluations with median values of 14 and 10, respectively. However, no significant differences were observed (*P* = 0.457).

**Fig. 3:**
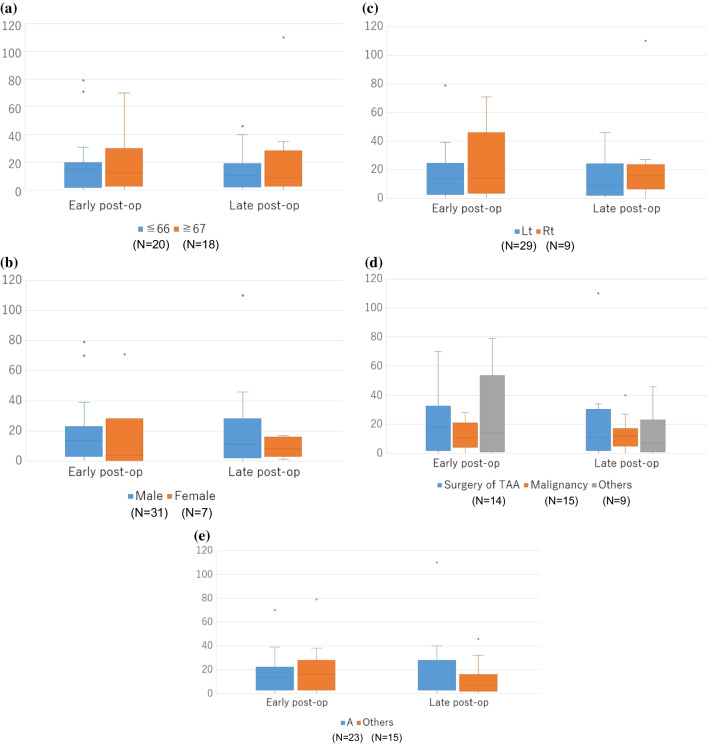
Comparison between early and late postoperative VHI for subscales grouping. a, age differences; b, sex; c, affected side; d, causes of UVFP; e, surgeons. VHI, voice handicap index

Five subscales, including age differences, sex, causes of UVFP, affected side, and surgeons, were analyzed (Fig. 3a–e). To analyze age differences, 38 patients eligible for comparison were divided into two groups: those aged 66 years or younger and those aged 67 years or older, with a median age of 66 years as the boundary. To analyze the causes of UVFP, patients were divided into three groups: surgery for TAA, malignancy, and others. For the analysis of surgeons, patients were divided into two groups comprising one surgeon (A) and other surgeons (B, C, and D). None of these groups showed significant differences between the early and late postoperative evaluations on each subscale, and none showed significant differences at each time point.

## Discussion

For normal voice production, appropriate glottal closure is required with the median location of the vocal folds, symmetrical vocal fold tension and masses, and supple mucosa [5, 15]. In patients with UVFP, incomplete glottic closure and reduced vocal fold tension cause problems with phonation; therefore, the principle of several interventions for UVFP is to medialize the paralyzed vocal fold and reduce the glottic opening to improve vocal efficiency during phonation [19]. Although vocal fold medialization can be performed with either the ML or IL method and generally produces comparable voice improvements, we experienced unfavorable results in patients with UVFP with severe breathy hoarseness due to a large posterior gap or vertical height difference in the vocal folds, as observed using a laryngeal flexible fiberscope. Hiramatsu et al. reported that the arytenoid cartilage on the affected side was pushed by the contralateral arytenoid and dislocated passively in the lateral and superior directions during phonation in three-dimensional computed tomography (3DCT) studies [20, 21]. They also conducted 3DCT in patients with UVFP with unsatisfactory outcomes after surgery and found that the posterior parts of the vocal folds were at different levels because the flaccid paralyzed arytenoid cartilage glided dorsocranially during phonation in patients with UVFP who underwent ML or IL [20]. From the findings on the movement of the paralyzed vocal folds, Tokashiki et al. emphasized the importance of eliminating this passive movement so that the vocal folds can develop strong resistance to exhaled airflow and push from the healthy side [22]. We performed MT alone or IL, using collagen or autologous fat, for patients with UVFP who presented with mild breathy hoarseness and a smaller glottal gap during phonation. However, we believe that AA is necessary for patients presenting with a wide glottal gap and vocal fold height mismatch during phonation. In combination with AA, ML using Gore-Tex^®^ is commonly used to compensate for vocal fold muscle atrophy.

To date, most reported postoperative outcomes of AA in patients with UVFP have been evaluated within or approximately a year. Chang et al. reported the postoperative outcomes of ML with AA at least 6 weeks after surgery, a relatively short-term evaluation, while confirming the resolution of postoperative edema [23]. Mortensen et al. demonstrated significant improvements in ML with AA in acoustic and aerodynamic parameters postoperatively at approximately 3 months [6]. Mes et al. assessed the voice outcomes of MT + AA using the VHI, perceptual evaluation, and aerodynamic evaluation 12 months postoperatively [24]. There have been few reports on the results of AA more than 2 years postoperatively. Hassan et al. focused on the priority of reinnervation of the thyroarytenoid muscle achieved by nerve-muscle pedicle (NMP) flap implantation [15]. In a comparison of AA ± TP1 and AA ± NMP, the AA ± NMP group showed steady improvement over the 2-year follow-up, which did not occur in the AA ± TP1 groups. Similarly, Kodama et al. reported excellent vocal outcomes of NMP with AA on vocal fold vibration, aerodynamic analysis, and perceptual evaluation postoperatively at 2-years [25]. However, their primary goal was to emphasize the significance of NMP and not to evaluate AA stability. This study is the first to evaluate long-term voice evaluation for more than 2 years after AA, with a median of approximately 5 years, and our results could offer stable and lasting voice improvement over the years. Furthermore, the characteristics or background of patients with UVFP possibly would not affect the long-term outcomes of AA surgery. In addition, minor differences in surgical technique by each surgeon, such as whether to cut the cricothyroid joint, preserve or remove the cartilage window during ML, and differences in surgical experience, did not affect long-term outcomes. This is a meaningful result because the AA procedure is a well-established surgical technique.

Of the 77 patients with UVFP who underwent AA, 6 (7.8%) underwent additional surgery. Of the 38 patients eligible for comparison between the early and late postoperative evaluations, 4 underwent additional surgery; there was no revision surgery for AA. The VHI values of the four patients in the late postoperative evaluation were relatively higher (3, 34, 35, and 46); however, there was no significant difference between the early and late evaluations (data not shown). As for the properties of Gore-Tex^®^ implant, Benninger et al. stated that compression of the Gore-Tex^®^ can occur over time while reducing the quality of voice [26]. Song et al. suggested that the mass effect exerted by the Gore-Tex^®^ implant may decrease over time because 13.7% of their patients required touch-up injection medialization after 54.7 months [27]. They attributed this to implant migration or compression and/or the natural loss of vocal fold bulk with aging. Furthermore, Siu et al. postulated the progressive fading of MT results due to possible continued vocal fold atrophy over the years [14]. Hassan et al. indicated that postoperative vocal fold atrophy, an inevitable outcome of the denervated thyroarytenoid muscle, causes the vocal fold bulk or mass to decrease over time [15]. For these reasons, we consider that AA has a stable and lasting effect on patients with UVFP. However, compression of the Gore-Tex^®^ implant used for ML and/or the progression of vocal fold atrophy over time, whether on the affected or unaffected side, may be reasons for the need for additional surgery.

The limitations of this study should be considered. First, a smaller number of patients were eligible for the comparison between the early and late postoperative evaluations. This was partly because 12 patients died or were terminally ill, and that there were 24 non-respondents, including 5 patients with unknown addresses, among the 66 patients who were asked to return the VHI questionnaire by mail. A long lapse in time after surgery may reduce the patient’s motivation to respond to the return request. Second, we used only the VHI self-assessment questionnaire for postoperative evaluation. The postoperative VHI is a relatively independent measurement parameter in patients with UVFP who underwent AA [28]. Franco et al. indicated that objective data, such as acoustic and aerodynamic measures, were effort-dependent and that these may not be reliable tools for measuring postsurgical voice outcomes; thus, patient surveys, including the VHI, maybe the best tools to measure the outcomes of surgical intervention [29]. In addition, Daniero et al. indicated that patients’ subjective assessments are critical and are the ultimate arbiter of success [8]. Although the VHI is the most widely available and reliable measure for patients with perceived voice disability, other vocal function examinations for a very long postoperative period would have confirmed the result.

## Conclusion

Based on our findings, we conclude that patients with UVFP who underwent AA surgery achieved stable voice improvement over the long term after surgery, regardless of the patient’s background. Patients with UVFP continue to live; thus, it is of great significance to demonstrate long-lasting voice improvement after AA surgery.

## Data Availability

Not applicable.
