# Correction: Long-term voice evaluation after arytenoid adduction surgery in patients with unilateral vocal fold paralysis

**DOI:** 10.1007/s00405-023-08422-x

**Published:** 2023-12-30

**Authors:** Kenichi Watanabe, Ai Hirano, Yuta Kobayashi, Takeshi Sato, Yohei Honkura, Yukio Katori

**Affiliations:** 1 Department of Otolaryngology, Tohoku Rosai Hospital, 4-3-21 Dainohara, Aoba-ku, Sendai, Miyagi 981-8563 Japan; 2 Department of Otolaryngology, Head and Neck Surgery, Tohoku University Graduate School of Medicine, 1-1 Seiryo-Machi, Aoba-ku, Sendai, Miyagi 980-8574 Japan

**Correction: European Archives of Oto-Rhino-Laryngology (2023) 280:5011–5017** 10.1007/s00405-023-08165-9

The article Long-term voice evaluation after arytenoid adduction surgery in patients with unilateral vocal fold paralysis, written by Kenichi Watanabe, Ai Hirano, Yuta Kobayashi, Takeshi Sato, Yohei Honkura, Yukio Katori was originally published Online First without Open Access. After publication in volume 280, issue 11, pages 5011–5017 the author decided to opt for Open Choice and to make the article an Open Access publication. Therefore, the copyright of the article has been changed to © The Author(s) 2023 and the article is forthwith distributed under the terms of the Creative Commons Attribution 4.0 International License, which permits use, sharing, adaptation, distribution and reproduction in any medium or format, as long as you give appropriate credit to the original author(s) and the source, provide a link to the Creative Commons licence, and indicate if changes were made. The images or other third party material in this article are included in the article’s Creative Commons licence, unless indicated otherwise in a credit line to the material. If material is not included in the article’s Creative Commons licence and your intended use is not permitted by statutory regulation or exceeds the permitted use, you will need to obtain permission directly from the copyright holder. To view a copy of this licence, visit http://creativecommons.org/licenses/by/4.0/.

The original article was updated.

